# Poisoning as a Profession

**Published:** 1898-02

**Authors:** 


### POISONING AS A PROFESSION.

In a recent number of the *Indian Lancet* appears an extensive article on the above subject. The writer, after making a few general remarks, tells us that in the early Christian era so many took up this calling that it was easy to secure the services of a professional poisoner. Indeed, the administration of poison became so general in the higher ranks of life that there was danger in partaking of food or drink at the tables of friends or the nearest relatives. During the period called the "dark age of Europe," professional poisoners became very numerous, some of whom were called men and women of the baser sort, as distinguished from their more prosperous fellows; to the author, however, there seems to be no distinction—they were all men and women of the baser sort. So greatly did poisoning increase and it must have been so lucrative as a profession that, from the fifteenth to the seventeenth century, two great criminal schools flourished in Venice and Italy, where the art was taught as a profession.

Early in the fifteenth century the Venetian school of poisoning became famous and produced a mania for poisoning, and to such a height did this mania rise, that the government of the States of Venice gravely considered the matter, and formally adopted and recognized the secret assassination by poison, and considered it a potent and useful agent for the removal of emperors, princes, and nobles who might be inimical to the government. A little consideration of the matter, says the writer, will show that it was not surprising that the mania should have reached this height, for we know, even in our own time, what a powerful force a popular mania is, and how uncontrollable it becomes when once it breaks bounds. There is the morphia mania of our day which, unless it can be restrained, will become the scourge of mankind; then, there are many popular harmful manias whose recent growth has been so great as to be a menace to our race.

Admitting the fact that the development and growth of popular manias are, as it were, ordinary occurrences of human life, it then becomes easier to understand the remarkable attitude of the government of the States of Venice towards secret poisoning. The notorious Council of Ten used to meet and consider the plans proposed for the removal of individuals obnoxious to the government. The account and record of their proceedings still exists, and exists in detail, giving the number of those who voted for and those who voted against the proposed murders; the reasons for the assassination are given, and even the sum is stated which was paid the professional poisoner and the individual who committed the crime. The senators treated the whole matter in an ironical spirit, for when the deed was accomplished and reported, they registered the murder by writing on the margin of their official record the single word "Factum."

It was generally known at the time in Venice that the government adopted the use of poison in removing obnoxious individuals, so John of Ragubo, a Franciscan brother, presents himself before the Council with an offer of a selection of poisons, declaring himself ready to kill any person they might desire to have put out of the way. John of Ragubo was not only a master poisoner, but a master villain; the cool audacity in which he stated his terms was remarkable. He asked for a pension of 1,500 ducats a year to be paid after the death of the first person he poisoned successfully; and he goes on to bargain with the Council, in the same calm, methodical way, for an increase to the annual sum of the pension for each successive individual that he murdered. This cool, matter-of-fact manner did not startle the members before whom he made his diabolical proposals, for the two Presidents,

Girilando Duoda and Pietro Guiarini, placed the matter before the Council, who, after discussion, resolved to accept it, characterizing it as a patriotic offer. John of Ragubo was told that the Emperor Maximillian was the first individual he should kill by poison. This John presented a curious tariff to the Council, as follows:

For the Great Sultan, 500 ducats.

For the King of Spain, 150 ducats, including the expenses of the journey.

For the Duke of Milan, 60 ducats.

For the Marquis of Mantua, 50 ducats.

For the Pope, 100 ducats.

John of Ragubo evidently considered the whole matter as a legitimate business, for he adds: "The further the journey, the more eminent the man, the more it is necessary to reward the toil and hardship undertaken, and the heavier must be the payment." G.

*American Medical Surgical Bulletin.*

